# CRMP2 Participates in Regulating Mitochondrial Morphology and Motility in Alzheimer’s Disease

**DOI:** 10.3390/cells12091287

**Published:** 2023-04-29

**Authors:** Tatiana Brustovetsky, Rajesh Khanna, Nickolay Brustovetsky

**Affiliations:** 1 Department of Pharmacology and Toxicology, Indiana University School of Medicine, 635 Barnhill Drive, Medical Science Building, Room 362, Indianapolis, IN 46202, USA; tbrousto@iupui.edu; 2 Department of Molecular Pathobiology, New York University, New York, NY 10010, USA; rk4272@nyu.edu; 3 College of Dentistry, NYU Pain Research Center, New York University, New York, NY 10010, USA; 4 Department of Neuroscience and Physiology and Neuroscience Institute, School of Medicine, New York University, New York, NY 10010, USA; 5 Stark Neurosciences Research Institute, Indiana University School of Medicine, Indianapolis, IN 46202, USA

**Keywords:** CRMP2, Alzheimer’s disease, cortical neurons, mitochondrial morphology, mitochondrial motility, neuronal cell death

## Abstract

Mitochondrial bioenergetics and dynamics (alterations in morphology and motility of mitochondria) play critical roles in neuronal reactions to varying energy requirements in health and disease. In Alzheimer’s disease (AD), mitochondria undergo excessive fission and become less motile. The mechanisms leading to these alterations are not completely clear. Here, we show that collapsin response mediator protein 2 (CRMP2) is hyperphosphorylated in AD and that is accompanied by a decreased interaction of CRMP2 with Drp1, Miro 2, and Mitofusin 2, which are proteins involved in regulating mitochondrial morphology and motility. CRMP2 was hyperphosphorylated in postmortem brain tissues of AD patients, in brain lysates, and in cultured cortical neurons from the double transgenic APP/PS1 mice, an AD mouse model. CRMP2 hyperphosphorylation and dissociation from its binding partners correlated with increased Drp1 recruitment to mitochondria, augmented mitochondrial fragmentation, and reduced mitochondrial motility. (S)-lacosamide ((S)-LCM), a small molecule that binds to CRMP2, decreased its phosphorylation at Ser 522 and Thr 509/514, and restored CRMP2′s interaction with Miro 2, Drp1, and Mitofusin 2. This was paralleled by decreased Drp1 recruitment to mitochondria, diminished mitochondrial fragmentation, and improved motility of the organelles. Additionally, (S)-LCM-protected cultured cortical AD neurons from cell death. Thus, our data suggest that CRMP2, in a phosphorylation-dependent manner, participates in the regulation of mitochondrial morphology and motility, and modulates neuronal survival in AD.

## 1. Introduction

Alzheimer’s disease (AD) is an incurable neurodegenerative disorder characterized by synaptic defects and neuronal degradation, particularly in the cortex and hippocampus [1,2]. AD is a common cause of dementia and a primary cause of morbidity and mortality in aging. Numerous studies linked AD to the intracellular accumulation of toxic amyloid-β peptides (Aβ) and the formation of extracellular Tau tangles [3,4]. Yet, a mechanistic link between these events, synaptic deficits, and neuronal degradation remains unclear.

Mitochondrial bioenergetics and dynamics (alterations in morphology and motility of mitochondria) play essential roles in neuronal reactions to varying energy needs in health and disease [5]. Altered bioenergetics, increased fission, decreased mitochondrial motility, and synaptic deficits have been reported in AD [6,7,8,9,10], and it has been proposed that defects in mitochondrial bioenergetics and dynamics are among the major contributors to AD pathogenesis [11,12,13,14]. Indeed, alterations in mitochondrial functions over time may lead to synaptic deficits and neuronal degradation [7,8]. However, the exact mechanisms leading to mitochondrial abnormalities in AD are not completely understood.

CRMP2 is an abundant cytosolic phosphoprotein that interacts with various proteins, regulating their activity and/or position [15,16,17,18,19,20,21]. As a result, CRMP2 plays a role in regulating diverse physiological processes, including mitochondrial dynamics [17,18]. Out of the five members of the CRMP family, CRMP2 maintains a high level of expression in adulthood [22]. CRMP2 is a substrate for glycogen synthase kinase-3β (GSK-3β) and cyclin dependent kinase 5 (Cdk5) [23,24,25,26], which have increased activity in AD [27,28,29,30]. Phosphorylation of CRMP2 at residues Thr 509 and Thr 514, targeted by GSK-3β, and Ser 522, targeted by Cdk5, is high in human AD brains [20,23,31,32,33]. CRMP2 hyperphosphorylation was also found in brain tissues of APP/PS1 and Tg2576 AD mice [20,32,34]. Increased CRMP2 phosphorylation in AD mice occurs prior to the onset of pathology, suggesting that hyperphosphorylation of CRMP2 is a very early process in AD [32]. In healthy brains, 30–50% of neuronal CRMP2 is phosphorylated at Thr 509/514, suggesting CRMP2 phosphorylation in AD may be reaching its maximum [33]. Consequently, CRMP2 is considered an emerging target in AD [35]. Still, the role of CRMP2 hyperphosphorylation in neuronal degradation and the progression of AD have not been fully addressed.

CRMP2 binds to mitochondria [17,36] and interacts with Drp1 and Miro 2 [17,18], which are proteins involved in mitochondrial fission [37] and motility [38], respectively. In addition, CRMP2 interacts with Kinesin 1 light chain (KLC1) [17,19] and Dynein [21]: motor proteins involved in mitochondrial traffic. CRMP2 hyperphosphorylation disrupts the CRMP2 interaction with Drp1 and Miro 2 [17,18]. These events are accompanied by increased mitochondrial fission, suppressed mitochondrial motility, and decreased neuronal viability [18]. Yet, it is unknown whether changes in CRMP2 phosphorylation and CRMP2 dissociation from its binding partners are linked to alterations in mitochondrial dynamics in AD and, subsequently, to AD pathology.

In the present study, we evaluated the CRMP2 phosphorylation state in postmortem brain tissues from AD patients and non-AD individuals, in brain lysates, and in cultured cortical neurons from the double transgenic APP/PS1 mice and their wild-type (WT) littermates. We explored the connection between the CRMP2 phosphorylation state and mitochondrial morphology and motility in cultured cortical neurons from APP/PS1 and WT mice. We explored the ability of a small molecule ((S)-2-acetamido-N-benzyl-3-methoxypropionamide, (S)-lacosamide, ((S)-LCM), which selectively suppresses CRMP2 phosphorylation at Ser 522 and Thr 509/514 by Cdk5 and GSK-3β kinases [39,40], to preserve CRMP2 interaction with its binding partners, prevent alterations in mitochondrial morphology and motility, and protect mouse AD neurons from cell death.

## 2. Materials and Methods

### 2.1. Human Postmortem Brain Tissues

Deidentified human postmortem cortical brain tissues were obtained from the University of Maryland Brain and Tissue Bank (UMDBTB) and the Icahn School of Medicine Mount Sinai Neuropathology Brain Bank, which are Brain and Tissue Repositories of the NIH NeuroBioBank. Anonymized information regarding samples of postmortem brains is presented in Supplementary Table S1. The specimens were stored at −86 °C. Before the tests, specimens were incubated in ice-cold medium containing 150 mM NaCl, 1 mM EDTA, 50 mM Tris–HCl, pH 7.4, and supplemented with Phosphatase and Protease Inhibitor Cocktails (Roche, Cat # 04906845001 and Cat # 04693124001) and gradually defrosted at 0 °C.

### 2.2. Animals

Experimental manipulations with mice were conducted according to the US National Institutes of Health Guide for the Care and Use of Laboratory Animals and according to the Indiana University School of Medicine Institutional Animal Care and Use Committee approved protocol (#20156 MD/R). The double transgenic APP/PS1 mice [41] (B6;C3Tg(APPswe, PSEN1dE9)85Dbo/Mmjax, Jackson Laboratory, MMRRC Strain # 034829-JAX) and wild-type (WT) genetic background C57BL/6;C3H mice (Jackson Laboratory) of both sexes were obtained from the Jackson Laboratory (Bar Harbor, ME, USA) and breeding mouse groups were housed in the Laboratory Animal Resource Center at Indiana University School of Medicine, Indianapolis, IN, USA. APP/PS1 mice were chosen because they have (i) hyperactivated Cdk5 and GSK-3β kinases, (ii) hyperphosphorylated CRMP2, (iii) suppressed mitochondrial traffic, and (iv) excessively fragmented mitochondria [8,10,42]. Hemizygous male APP/PS1 mice were bred with female C57BL/6;C3H mice. The mice were kept under normal conditions with *ad libitum* access to food and water. Mice were kept in cages made out of polycarbonate, 3 mice per cage.

### 2.3. Genotyping

Mouse pups were genotyped using a PCR test with tail DNA. Briefly, PCR of tail DNA was performed according to the protocol offered by the Jackson Laboratory utilizing oligonucleotide primers oIMR3610 (AGG ACT GAC CAC TCG ACC AG) and oIMR3611 (CGG GGG TCT AGT TCT GCA T) for APP and oIMR1644 (AAT AGA GAA CGG CAG GAG CA) and oIMR1645 (GCC ATG AGG GCA CTA ATC AT) for PSEN1, purchased from Invitrogen (Carlsbad, CA, USA). The PCR reaction mixture included 1 µL DNA template and 23 µL Platinum PCR SuperMix (Invitrogen) complemented with 0.39 µM of each primer (Invitrogen); the entire volume was 25 µL. The following cycling conditions were employed: 5 min at 95 °C, 35 cycles at 30 s at 95 °C, 30 s at 56 °C, 60 s at 72 °C, and 10 min at 72 °C. Reaction products were evaluated on a 1.2% agarose gel subjected to 100 V for 60 min using Tris–acetate–EDTA running buffer, including 1× GelRed^TM^ Nucleic Acid Gel Stain (Biotium, Fremont, CA, USA).

### 2.4. Isolation and Purification of Brain Synaptic Mitochondria

Brain synaptic (neuronal) mitochondria were isolated from mouse cortices and purified using a continuous 30% Percoll gradient as described [17,43].

### 2.5. Isolation of Mitochondria from Cultured Neurons

Mitochondria from mouse cultured cortical neurons were isolated as described previously [44] with some modifications. Neurons were plated on 60 mm Petri dishes and cultured for 12–14 DIV as described above. To isolate mitochondria, the Petri dishes with neuronal cultures were washed twice with ice-cold calcium- and magnesium-free phosphate-buffered saline containing 1 mM EGTA. Cells were transferred into 0.8 mL (per dish) of ice-cold isolation buffer containing 225 mM mannitol, 75 mM sucrose, 1 mM EGTA, 10 mM HEPES–KOH, pH 7.4, and 0.1% fatty acid-free bovine serum albumin (MP Biomedicals, Irvine, CA, USA). Then, cells were combined and homogenized using a Dounce-type homogenizer (10 passes with a loose A pestle followed by 20 passes with a tight pestle B). The homogenates were centrifuged for 10 min at 1000× *g* at 4 °C, and the supernatant was transferred into a fresh tube. The remaining pellet was resuspended in the isolation buffer and centrifuged at 1000× *g* for 5 min. The first and second supernatants were combined and centrifuged at 15,000× *g* for 12 min at 4 °C. The mitochondrial pellet was resuspended in the isolation buffer with 0.1 mM EGTA but without albumin, and centrifuged at 15,000× *g* for 12 min. All isolation procedures were performed on ice. Usually, twelve 60 mm Petri dishes with neurons were used for one mitochondrial preparation.

### 2.6. Neuronal Cell Culture

Cell cultures of mouse cortical neurons were generated from brain cortices of postnatal day 1 (P1) APP/PS1 and WT mice in accord with the protocol approved by IACUC and techniques described earlier [45]. For immunoblotting and co-immunoprecipitation (co-IP) experiments neurons were plated at 200,000 cells per 35 mm Petri dish. For assessment of mitochondrial morphology and motility and evaluation of spontaneous neuronal cell death, neurons were plated at a lower density (10,000 cells) per glass-bottom (10 mm diameter) Petri dish to decrease the likelihood of neuronal clumping. For all neuronal platings, 35 μg/mL uridine supplemented with 15 μg/mL 5-fluoro-2′-deoxyuridine was applied 24 h following plating to suppress proliferation of microglia. Neuronal cultures were kept in a 5% CO_2_ atmosphere at 37 °C in MEM complemented with 10% NuSerum (BD Bioscience, Bedford, MA, USA) and 27 mM glucose. Experiments were conducted on cell cultures at 12–14 day in vitro (12–14 DIV). For cell death assessment, cell cultures were grown on glass-bottom 35 mm Petri dishes for 21 DIV.

### 2.7. Western Blotting

Specimens of postmortem brain tissues (prefrontal cortex) from AD patients and non-AD individuals were emulsified in a medium containing 150 mM NaCl, 50 mM Tris–HCl, pH 7.4, 1 mM EDTA, 1% NP-40, 0.1% SDS, and complemented with Phosphatase and Protease Inhibitor Cocktails (Roche, Indianapolis, IN, USA, Cat # 04906845001 and Cat # 04693124001). The obtained mixtures were kept for 30 min at 0 °C and then centrifugated at 100,000× *g* 30 min. The pellet was dumped and the supernatant was utilized for polyacrylamide gel electrophoresis (PAGE). Mouse cortical neurons in culture were prepared for PAGE in a manner analogous to specimens of postmortem brains. Bis–Tris gels (4–12%, Invitrogen, Carlsbad, CA, USA, Cat # NP0335) were utilized to segregate proteins by PAGE (20 µg protein per lane). After PAGE, proteins were translocated to a Hybond-ECL nitrocellulose membrane (Amersham Biosciences, Piscataway, NJ, USA, Cat # RPN78D) and kept at 22 °C for an hour in a blocking solution comprised of either phosphate-buffered saline, pH 7.2, containing 5% BSA and 0.15% Triton X-100, or phosphate-buffered saline, pH 7.2, containing 5% milk and 0.15% Triton X-100 for total protein Western blotting. For phosphoprotein Western blotting, Tris–HCl-adjusted saline, pH 7.2, containing 5% BSA and 0.15% Triton X-100 was utilized. Following incubation in the blocking solution, blots were kept with either sheep anti-CRMP2 pThr 509/514 (Kinasource, Dundee, UK, Cat # PB-043, 1:1500), rabbit anti-CRMP2 pSer522 (ECM Biosciences, Versailles, KY, USA, Cat # CP2191, 1:1500), mouse anti-GAPDH (Abcam, Cambridge, MA, USA, Cat # ab9484, 1:2000), rabbit anti-Drp1 pSer 616 (Cell Signaling, Danvers, MA, USA, Cat # 3455, 1:1000), rabbit anti-CRMP2 (Sigma, St. Louis, MO, USA, Cat # C2993, 1:1000), mouse anti-β-actin (ThermoFisher Scientific, Carlsbad, CA, USA, Cat # MA5-15739, 1:1000), or rabbit anti-Miro 2 (Proteintech, Rosemont, IL, USA, Cat # 11237-1-AP, 1:1000), rabbit anti-Mitofusin-2 (Sigma, Cat # M6319), and rabbit anti-DRP1 (Santa Cruz Biotechnology, Paso Robles, CA, USA, Cat # sc-32898, 1:100) antibodies. Then, blots were incubated with either goat anti-rabbit or goat anti-mouse IgG (1:25,000 or 1:20,000, respectively) conjugated with horseradish peroxidase (Jackson ImmunoResearch Laboratories, West Grove, PA, USA) and visualized with Supersignal West Pico chemiluminescent reagents (Pierce, Rockford, IL, USA, Cat # 32106). Page Ruler Plus Prestained Protein Ladder (5 μL, Thermo Fisher; Cat # 26619), a molecular mass marker, was employed for molecular mass evaluation of the bands. The Western blotting images were processed, and the density of bands was assessed after subtracting background using Adobe Photoshop 22.2.0.

### 2.8. Co-Immunoprecipitation

Cultured cortical neurons (12–14 DIV) were incubated either with a vehicle (0.01% DMSO) or with 10 μM (S)-lacosamide for 7 days prior to the experiment. (S)-lacosamide, ((S)-2-acetamido-N-benzyl-3-methoxypropionamide, (S)-LCM)) was acquired from TLC Pharmaceutical Standards (Cat # L-2812, Newmarket, ON, Canada). Following treatment, cells were incubated in the lysis buffer, comprising 20 mM Tris–HCl, 139 mM NaCl, supplemented with Proteinase Inhibitor Cocktail (Roche), 0.1% SDS, and 1% NP40. Lysates were cleared to eliminate any precipitated residue using Protein A/G agarose beads (Santa Cruz Biotechnology, Cat # sc-2002, Santa Cruz, CA, USA) for 2 h on ice. Then, cell lysates were kept twelve hours with primary rabbit anti-Mitofusin-2 (Sigma, Cat # M6319), rabbit anti-Miro 2 (Proteintech, Cat # 11237-1-AP, 1:500), rabbit anti-DRP1 (Santa Cruz Biotechnology, Cat # sc-32898, 1:100), rabbit anti-CRMP2 (Sigma, Cat # C2993, 1:1000), mouse anti-MFF (mitochondrial fission factor, Santa Cruz Biotechnology, Cat # sc-398731, 1:500), rabbit anti-FIS1 (mitochondrial fission 1 protein, ThermoFisher Scientific, Cat # 10956-1-AP, 1:100), rabbit anti-syntabulin (ThermoFisher Scientific, Cat # 16972-1-AP, 1:500), or rabbit anti-syntaphilin (ThermoFisher Scientific, Cat # 13646-1-AP, 1:500) antibodies under gentle stirring on ice followed by treatment with Protein A/G agarose beads (Santa Cruz Biotechnology, Cat # sc-2002) for 2 h on ice. The immune-captured complexes were washed with lysis buffer three times prior to heating at 70 °C in identical volumes of SDS loading dye (Invitrogen, Carlsbad, CA, USA). In these tests, Tris–Acetate gels (3–8%, Invitrogen, Cat # EA0375BOX) were used to segregate proteins by PAGE (20 µg protein per lane). Then, Western blotting was used to process samples as reported earlier [46,47]. Western blots were tested with rabbit anti-Mitofusin 2, rabbit anti-Miro 2, rabbit anti-CRMP2, rabbit anti-Drp1, anti-FIS1, rabbit anti-MFF, rabbit anti-syntabulin, or rabbit anti-syntaphilin antibodies (1:1000 dilution for each). The shown Western blots are typical from at least 3 separate experiments. The images of Western blots were processed, and band densities were assessed following subtraction of background employing Adobe Photoshop (version 22.2.0).

### 2.9. Neuronal Transfection

To visualize mitochondria in live neurons, cells were transfected employing the electroporator BTX 630 ECM (Harvard Apparatus, Holliston, MA, USA) and using a plasmid that encodes mitochondrially targeted enhanced yellow fluorescent protein (mito-eYFP, generous gift from Dr. Roger Tsien, UCSD). In selected experiments, neurons were co-transfected with a plasmid encoding mito-eYFP and anti-CRMP2 siRNA (ACTCCTTCCTCGTGTACATTT) [48] or scramble siRNA. In these experiments, neurons with mito-eYFP had significantly downregulated CRMP2. The transfection technique, based on electroporation, resulted in an ~10% transfection rate in cultured cortical neurons. This transfection rate was not adequate for Western blotting or real-time PCR to verify CRMP2 downregulation. Accordingly, CRMP2 downregulation was analyzed using fluorescent microscopy and a single-cell analysis. The images of transfected neurons were captured at 12–14 days following transfection.

### 2.10. Mitochondrial Morphology

Morphology of mitochondria in cultured cortical neurons was evaluated at 23 °C as described earlier [17,49]. Briefly, serial stacks of mitochondrial images were captured employing spinning-disk confocal microscopy. In these experiments, an inverted microscope Nikon Eclipse TE2000-U outfitted with an Andor iXon^EM^+ DU-897 back-thinned EM-CCD camera (Andor Technology, South Windsor, CT, USA), a spinning-disk confocal unit Yokogawa CSU-10, and a Prior H-117 motorized flat-top stage (Prior Scientific, Rockland, MA, USA) was utilized. The software Andor iQ 1.4 (Andor Technology, South Windsor, CT, USA) was controlling this setup. To take images of mitochondria, cultured neurons were exposed to 488 nm utilizing a Kr/Ar air-cooled laser T643-RYB-A02 (Melles Griot, Carlsbad, CA, USA). The intensity of the laser was adjusted to the minimal value (<5%), which was appropriate for obtaining adequate quality images of mitochondria, yet preventing unnecessary photobleaching. Fluorescence was recorded using a 505 nm dichroic mirror and a 535 ± 25 nm emission filter with an objective Nikon CFI Plan Apo 100× 1.4 NA. The z-stacks of mitochondrial images were captured employing the piezoelectric positioning device PIFOC^®^ P-721 (Physik Instrumente, Auburn, MA, USA) with a z-step 0.1 μm. The 3D deconvolution of serial images (z-stacks) and 3D rendering was accomplished using AutoDeblur Gold CF 1.4.1 software (MediaCybernetics, Silver Spring, MD, USA). Three-dimensional maximal projections of mitochondrial images were obtained by employing Imaris 5.7.0 (Bitplane Inc., Saint Paul, MN, USA) as we reported earlier [49]. To adjust the protocol for the processing of images and assessment of mitochondrial length, fluorescent beads were utilized as described [49]. The shape (length) of individual organelles was assessed in neurites. One hundred randomly selected mitochondria from 10–12 neurons from at least 3 separate platings were evaluated. During these experiments, cells were kept in the solution comprising 3 mM KCl, 139 mM NaCl, 1.8 mM CaCl_2_, 0.8 mM MgCl_2_, 10 mM NaHEPES, pH 7.4, 65 mM sucrose and 5 mM glucose. Sucrose was utilized to keep osmolarity of the bath solution similar to osmolarity of the growth solution (340 mosm). Osmolarity was determined with the osmometer Osmette II™ (Precision Systems Inc., Natick, MA, USA).

### 2.11. Mitochondrial Motility

Mitochondrial traffic in cortical neurons in culture was evaluated at 37 °C employing wide-field time-laps fluorescence microscopy as described [17]. Mitochondrial motility was documented with an inverted microscope Nikon Eclipse TE2000-U utilizing Nikon CFI Plan Apo 100× 1.4 NA objective and Cool SNAP_HQ_ Photometrics camera (Roper Scientific, Tucson, AZ, USA) managed by MetaMorph software 6.3 (Molecular Devices, Downingtown, PA, USA). The excitation light (480 ± 20 nm) was provided by a Lambda-LS system (Sutter Instruments, Novato, CA, USA), and fluorescence was recorded with a 505 nm dichroic mirror at 535 ± 25 nm. The images were captured at 1 Hz during the whole duration of the experiment (5 min). Mitochondrial traffic was evaluated following construction of kymographs with NIH ImageJ software (version 1.53a).

### 2.12. Neuronal Cell Death

Neurons were plated onto glass-bottom (10 mm diameter) 35 mm Petri dishes and cultured in vitro for 21 days and then cell death was evaluated by Chromatin Condensation/Membrane Permeability/Dead Cell Apoptosis Kit (ThermoFisher Scientific, Cat # V23201), comprising Hoechst 33342 (nuclear marker), YO-PRO-1 (apoptosis marker), and propidium iodide (PI, necrosis marker). Cultured neurons from five different platings of three different types of cell cultures were used: (i) **WT** neurons, (ii) **AD** neurons treated with a vehicle (Veh, 0.01% DMSO) for seven days prior to cell death analysis, and (iii) **AD** neurons treated with 10 µM (S)-LCM for seven days before cell death assessment. With each cell culture, neurons were counted in a blind manner in two fields of view (each with 45–70 cells) and it was repeated with three different Petri dishes per each type of neuronal culture. Dying neurons were documented using an inverted microscope Nikon Eclipse TE2000-U (Nikon Instruments Inc., Melville, NY, USA) outfitted with a Nikon CFI SuperFluor 20× 0.75 NA objective and a CoolSNAP_HQ_ Photometrics camera (Roper Scientific, Tucson, AZ, USA) managed by MetaMorph software (version 6.3, Molecular Devices, Downingtown, PA, USA).

### 2.13. Statistics

Data are shown as mean ± SD of the specified number of independent experiments. We evaluated our data using SigmaPlot v15.1 (Inpixon, Palo Alto, CA, USA). This software analyses normality (Shapiro–Wilk method) and equal variance of data (Brown–Forsythe method). In some of our experiments these criteria were met, while in others failed. In the former case, data were analyzed by parametric one-way analysis of variance (ANOVA) followed by a Holm–Sidak test. In the latter case, we evaluated the statistical significance of the difference using a nonparametric Kruskal–Wallis ANOVA on ranks followed by a Tukey test. The description of statistical analysis is included in the Figure legends.

## 3. Results

### 3.1. Hyperphosphorylation of CRMP2 in AD

**Figure 1:**
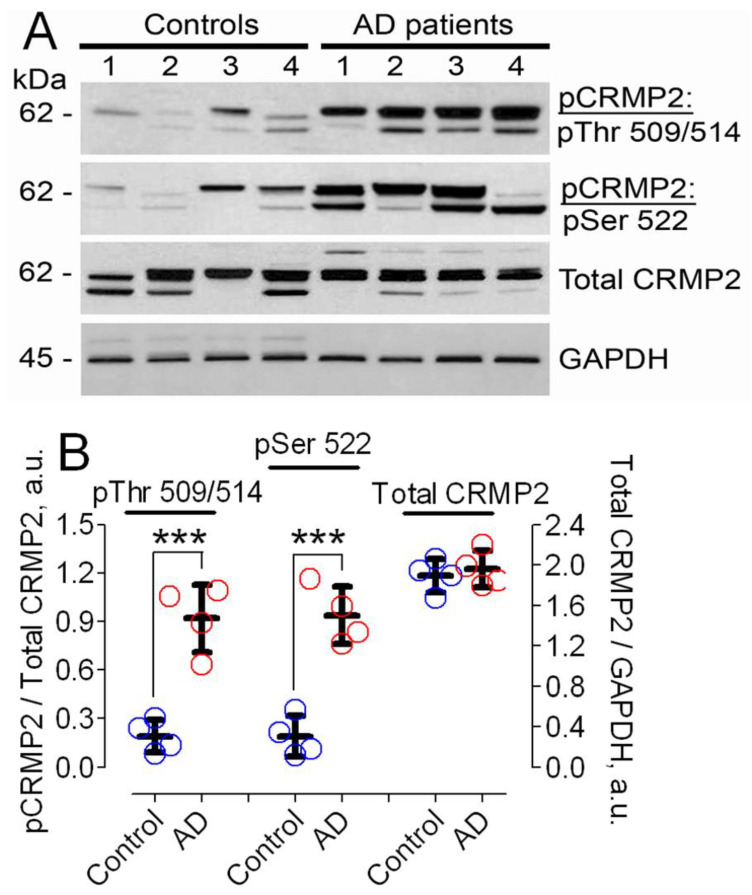
**CRMP2 is hyperphosphorylated at Ser 522 and Thr 509/514, but total CRMP2 is unchanged in postmortem brain tissues of AD patients (AD, red circles) compared to brain tissues from control non-AD individuals (Control, blue circles).** In (**A**), Western blotting of brain lysates from AD patients and unaffected individuals. Western blotting was performed with anti-pCRMP2 antibodies for pSer 522 and pThr 509/514, total CRMP2, and GAPDH as a loading control. Molecular weights (kilodalton, kDa) are shown on the left side of the blots. In (**B**), statistical summary of Western blotting data. Phosphorylation of CRMP2 at Ser 522 and Thr 509/514 was normalized per total CRMP2 expression and data are linked to the left Y-axis; a.u., arbitrary units. Total CRMP2 expression was normalized per GAPDH and data are linked to the right Y-axis. Data are mean ± SD, *** *p* < 0.001, N = 4. Data were analyzed by parametric one-way analysis of variance (ANOVA) with Holm–Sidak post-test.

Earlier, it was reported that CRMP2 is hyperphosphorylated in postmortem brain tissues of AD patients [20,32,33]. We confirmed this finding in our experiments (Figure 1). CRMP2 was hyperphosphorylated at Ser 522 and Thr 509/514, whereas total CRMP2 expression was without change.

**Figure 2:**
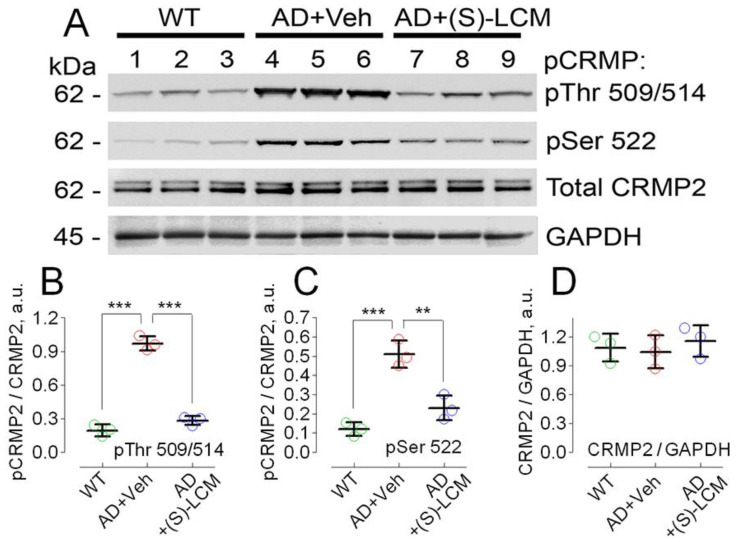
**CRMP2 is hyperphosphorylated at Ser 522 and Thr 509/514, but total CRMP2 is unchanged in lysates of brain cortices of APP/PS1 mice compared to lysates from WT littermates.** In (**A**), representative immunoblots. (S)-LCM significantly reduced CRMP2 phosphorylation. Lanes 1–3, WT mice; lanes 4–6, AD mice; lanes 7–9, AD mice treated with (S)-LCM. (S)-LCM was delivered by oral gavage (10 mg/kg body weight for 7 days prior to analysis). AD mice received a vehicle (Veh, 10 μL DMSO in 0.2 mL saline daily for 7 days before the test). In (**B**–**D**), statistical summaries. Green circles, wild-type (WT) mice; red circles, AD mice treated with a vehicle; blue circles, AD mice treated with (S)-LCM. Data are mean ± SD, ** *p* < 0.01, *** *p* < 0.001, N = 3 biological replicates. Data were analyzed by parametric one-way analysis of variance (ANOVA) with Holm–Sidak post-test.

It was also reported that CRMP2 is hyperphosphorylated in brains of double-transgenic APP/PS1 mice [32], and we also found that CRMP2 was hyperphosphorylated at Ser 522 and Thr 509/514 in lysates of cortices from 3-month old APP/PS1 mice (Figure 2).

It was recently discovered that (S)-lacosamide ((S)-LCM), a small molecule that is an inactive enantiomer of the anti-epileptic drug (R)-lacosamide (Vimpat^®^), can bind to CRMP2 and prevent its hyperphosphorylation [50]. Our experiments involved administering (S)-LCM to APP/PS1 mice (10 mg/kg body weight delivered by gavage for 7 days prior to the experiment), which led to a reduction in CRMP2 phosphorylation at Thr 509/514 and Ser 522 in cortical tissue lysates from the mice (Figure 2).

**Figure 3:**
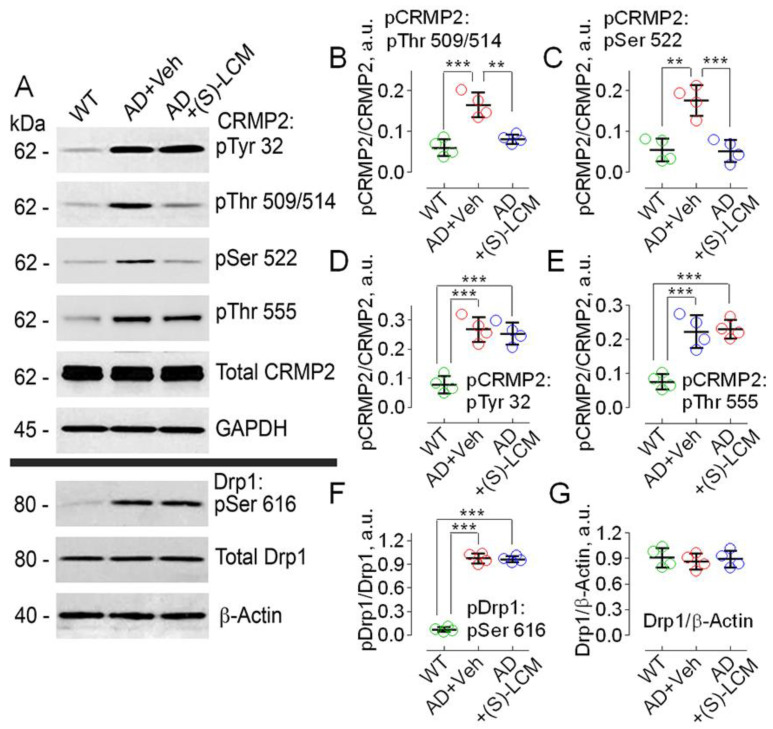
**Hyperphosphorylation of CRMP2 at Ser 522, Thr 509/514, Tyr 32, Thr 555, and hyperphosphorylation of Drp1 at Ser 616 in cultured cortical neurons from APP/PS1 mice compared to neurons from WT littermates. (S)-LCM (10 µM for 7 days) significantly reduced CRMP2 phosphorylation at Ser 522 and Thr 509/514, but not at Thr 555 and Tyr 32 or at Ser 616 of Drp1.** Cortical neurons were isolated from P1 APP/PS1 (AD) and WT mice of both sexes and cultured for 12–14 days in vitro (12–14 DIV). In (**A**), representative immunoblots. (**B**–**G**), statistical summaries based on densitometry data. Where shown, neurons were exposed to 10 µM (S)-LCM for the last 7 days before the experiments. β-actin and GAPDH are loading controls. Here and in other experiments with (S)-LCM applied to cultured neurons, 0.01% DMSO was used as a vehicle (Veh). Green circles, neurons from wild-type (WT) mice; red circles, neurons from AD mice treated with a vehicle; blue circles, neurons from AD mice treated with (S)-LCM. Data are mean ± SD, ** *p* < 0.01, *** *p* < 0.001, N = 4 separate experiments with neurons from different platings. Data were analyzed by parametric one-way analysis of variance (ANOVA) with Holm–Sidak post-test.

In the following experiments, we used cultured cortical neurons (12–14 DIV) from postnatal day 1 (P1) APP/PS1 mice. Primary neurons from embryonic or early postnatal AD mice are widely used in AD research and allow separation of AD pathology from normal aging [8,42,51]. In these experiments, we found CRMP2 hyperphosphorylation at Ser 522 and Thr 509/514, as well as at Tyr 32 and Thr 555 (Figure 3). Treatment of cultured cortical AD neurons with 10 μM (S)-LCM for 7 days significantly reduced CRMP2 phosphorylation at Ser 522 and Thr 509/514, but not at Thr 555 and Tyr 32. In addition, Ser 616 of Drp1 was hyperphosphorylated in AD neurons (Figure 3A,F), but (S)-LCM failed to attenuate Drp1 phosphorylation. Overall, our experiments confirmed CRMP2 hyperphosphorylation in AD and demonstrated that (S)-LCM antagonizes CRMP2 hyperphosphorylation.

### 3.2. CRMP2 Interaction with Proteins Participating in Regulation of Mitochondrial Morphology and Motility in AD Neurons

**Figure 4:**
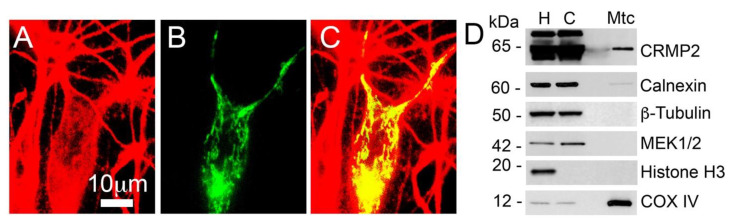
**CRMP2 colocalization with mitochondria in mouse cultured cortical neurons.** CRMP2 was stained with anti-CRMP2 antibody (**A**). Mitochondria were visualized using mitochondrially targeted enhanced yellow fluorescent protein (mito-eYFP) (**B**). Overlay of CRMP2 and mitochondrial images (**C**), yellow). In (**D**), CRMP2 is attached to synaptic mitochondria isolated from brain cortices of C57BL/6;C3H mice. **H**, homogenate; **C**, cytosol; **Mtc**, mitochondria. LAMP1, Calnexin, MEK1/2, COX IV, and Histone H3 are lysosomal, ER, cytosolic, mitochondrial, and nuclear markers, respectively. Cultured cortical neurons (14 DIV) were produced from postnatal day 1 (P1) wild-type C57BL/6;C3H mice, a background for APP/PS1 mice. Representative data from N = 5 biological repeats is shown.

In our recent study, we demonstrated that CRMP2 is co-localized with mitochondria in the somata of cultured striatal neurons and plays a role in regulating mitochondrial dynamics in these cells [17,18]. As cortical neurons are among the most affected cell types in AD and synaptic deficits are a major factor in AD pathology [1,2], it was important to investigate whether CRMP2 is also co-localized with mitochondria in cortical neurons and involved in synaptic mitochondria. Our present study showed that CRMP2 is indeed co-localized with mitochondria in cultured cortical neurons (Figure 4A–C). CRMP2 is a highly expressed cytoplasmic phosphoprotein in brain tissues, and the CRMP2 staining in Figure 4 accurately reflects its abundance in neurons, rather than being a result of over-exposure. This image reflects the actual abundance of CRMP2 in neurons. CRMP2 expression is spread across the cell, providing a strong basis for CRMP2–mitochondria interaction. In these experiments, mitochondria were stained with mitochondrially targeted enhanced yellow fluorescent protein (mito-eYFP). The genetic construct encoding mito-eYFP was delivered to neurons by electroporation. This technique provides an approximate transfection rate of 10% [52]. Correspondingly, in Figure 4B only one neuron was transfected and expressed mito-eYFP and, therefore, mitochondria were visible only in this transfected neuron. In addition to immunocytochemistry experiments, using immunoblotting, we detected CRMP2 in synaptic (neuronal) mitochondria (Figure 4D), isolated from mouse brain cortices and purified on a 30% continuous Percoll gradient as reported [17,43]. Thus, CRMP2 directly interacts with synaptic mitochondria in cortical neurons.

**Figure 5:**
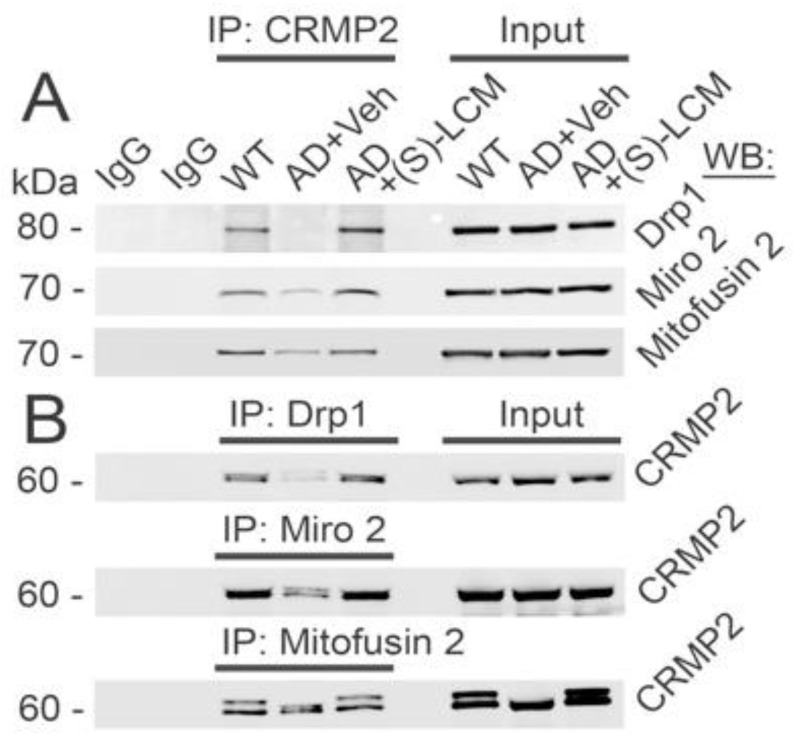
**CRMP2 interacts with Drp1, Miro 2, and Mitofusin 2 in cultured cortical neurons (14 DIV) from APP/PS1 (AD) and WT mice. In AD neurons, CRMP2 interaction with Drp1, Miro 2, and Mitofusin 2 was disrupted. (S)-LCM) precluded CRMP2 dissociation from these proteins.** In (**A**), immunoprecipitation (IP) of CRMP2 with Miro 2, Drp1, and Mitofusin 2 using pull-down procedure with anti-CRMP2 antibody followed by immunoblotting with anti-Miro 2, anti-Drp1, and anti-Mitofusin 2 antibodies. In (**B**), IP of Miro 2, Drp1, and Mitofusin 2 with CRMP2 using pull-down procedure with anti-Miro 2, anti-Drp1, and anti-Mitofusin 2 antibodies followed by immunoblotting with anti-CRMP2 antibody. Cells were exposed to either a vehicle (Veh, 0.01% DMSO) or 10 µM (S)-LCM for 7 days prior to analysis. Cortical neurons were isolated from P1 APP/PS1 (AD) and WT mice of both sexes and cultured for 12–14 days in vitro (12–14 DIV). For the input, 5% of total protein was used in the immunoprecipitation procedure. Representative data from N = 5 biological repeats is shown.

Next, we investigated the potential mitochondrial binding partners of CRMP2. Co-immunoprecipitation (co-IP) experiments with mouse cortical neurons in culture from APP/PS1 mice revealed that CRMP2 interacts with Drp1, Miro 2, and Mitofusin 2 (Figure 5) proteins involved in regulating mitochondrial motility and morphology [5].

The hyperphosphorylation of CRMP2 in AD neurons (Figure 3) resulted in a significant reduction in its interaction with binding partners (Figure 5). However, treatment with (S)-LCM (10μM in the growth medium for 7 days prior to the experiment) prevented the hyperphosphorylation of CRMP2 at Ser 522 and Thr 509/514 (Figure 3) and preserved its interaction with Miro 2, Drp1, and Mitofusin 2 (Figure 5). This demonstrates that CRMP2 interacts with proteins involved in regulating mitochondrial motility and morphology in a phosphorylation-dependent manner.

### 3.3. CRMP2 and Mitochondrial Motility and Morphology in AD Neurons

**Figure 6:**
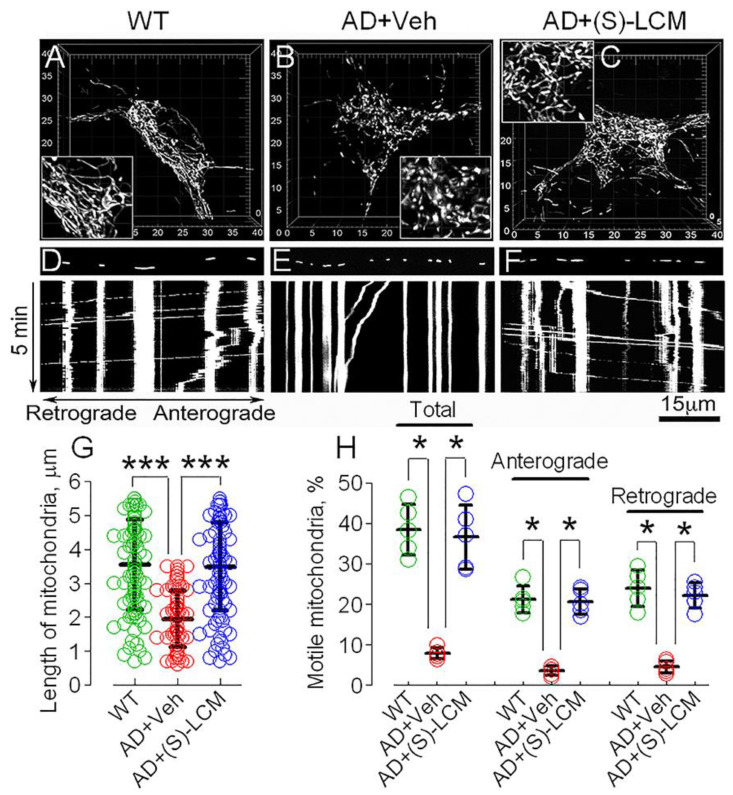
**Altered mitochondrial morphology and motility in cortical neurons in culture from APP/PS1 mice. (S)-LCM improved mitochondrial morphology and traffic.** In (**A**–**F**), mitochondria in cultured cortical neurons (12–14 DIV) visualized with mito-eYFP and imaged with a spinning-disk confocal microscopy. Mitochondrial morphology was analyzed using 3D reconstruction of serial images (z-stacks). In *Insets*, the organelles are demonstrated at doubled magnification. In (**A**), neuron from WT mice; in (**B**), neuron from APP/PS1 mice, these neurons were treated with a vehicle (Veh, 0.01% DMSO) for 7 days before imaging; in (**C**), APP/PS1 neuron incubated with 10 μM (S)-LCM for 7 days before imaging; in (**D**–**F**), mitochondrial traffic in neurites of WT neuron (**D**), APP/PS1 neuron treated with a vehicle (Veh, 0.01% DMSO) for 7 days before imaging (**E**), and APP/PS1 neuron treated with 10 μM (S)-LCM for 7 days before imaging (**F**). Here, and in Figure 7, vertical traces represent immobile mitochondria, angled traces represent trafficking organelles. The bands with mitochondrial fluorescent images demonstrate the organelles at the beginning of experiments. Motility of mitochondria was documented for five minutes at 37 °C. In (**G**), the length of neuronal mitochondria in µm. Here, and in Figure 7, one hundred randomly selected mitochondria from 10–12 cells from three different platings were assessed employing Imaris Measurement Pro 6.4 software (Bitplane, South Windsor, CT, USA) [17,18,49]. In (**H**), fractions of moving organelles and mitochondria trafficking in retrograde and anterograde directions shown as percentage of total number of analyzed mitochondria. Green circles, neurons from wild-type (WT) mice; red circles, neurons from AD mice treated with a vehicle; blue circles, neurons from AD mice treated with (S)-LCM. Data are mean ± SD, * *p* < 0.05, *** *p* < 0.001, N = 5 separate experiments with neurons from different platings. Data were analyzed by nonparametric Kruskal–Wallis ANOVA on ranks followed by Tukey test.

CRMP2 hyperphosphorylation (Figure 3) and a reduced binding of CRMP2 to Miro 2, Drp1, and Mitofusin 2 (Figure 5) in cortical neurons from APP/PS1 mice were accompanied by augmented mitochondrial fragmentation and suppressed mitochondrial traffic (Figure 6).

In addition to preventing CRMP2 hyperphosphorylation (Figure 3) and preserving CRMP2 binding to Miro 2, Drp1, and Mitofusin 2 (Figure 5), (S)-LCM restored mitochondrial motility and morphology in AD neurons (Figure 6). In our experiments, (S)-LCM alone applied to WT neurons did not cause detectable changes in mitochondrial morphology or motility (Supplementary Figure S6). Since (S)-LCM prevented hyperphosphorylation of CRMP2, but not Drp1 (Figure 3), the (S)-LCM effect on mitochondrial motility and morphology most likely was due to CRMP2 dephosphorylation and not due to changes in phosphorylation of Drp1.

**Figure 7:**
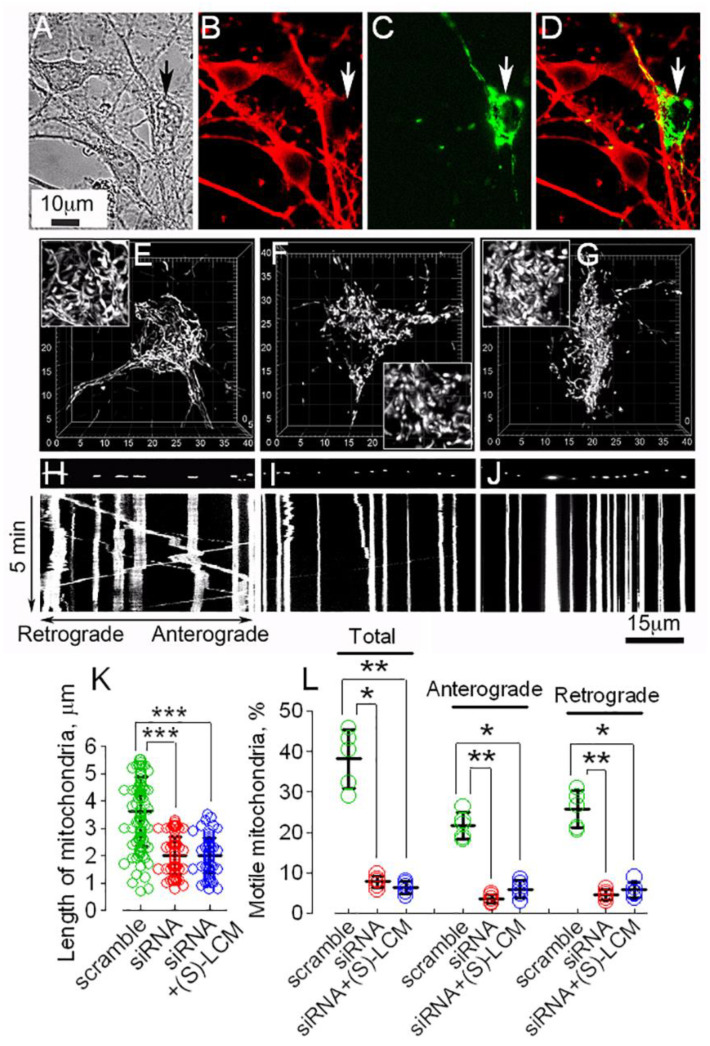
**CRMP2 ablation using siRNA-augmented mitochondrial fragmentation and reduced mitochondrial motility in cultured cortical neurons (12–14 DIV) from WT mice.** The results of representative experiments with cultured cortical neurons are shown. Neurons were isolated from P1 wild-type C57BL/6;C3H mice. In (**A**), phase-contrast bright-field image. In (**B**–**D**), typical immunochemistry staining is shown. (**B**), CRMP2 immunostaining. (**C**), visualization of mitochondria with mito-eYFP. (**D**), overlapped mitochondria and CRMP2 images. *Arrows*, neuron with successful transfection. Delivery of anti-CRMP2 siRNA (ACTCCTTCCTCGTG TACATTT) led to ablation of CRMP2 (**B**). In (**E**–**G**), mitochondrial morphology in cultured cortical neurons is shown. In (**E**), scramble siRNA did not change mitochondrial morphology. In (**F**), anti-CRMP2 siRNA resulted in an increased mitochondrial fission. In (**G**), a neuron with deleted CRMP2 and treated with 10 μM (S)-LCM for 7 days; mitochondria remained fragmented. Mito-eYFP was used to visualize mitochondria in live neurons. Mitochondrial morphology was assessed employing z-stacks of serial images and 3D reconstruction. In (**H**–**J**), representative kymographs illustrating mitochondrial motility are shown. Mitochondrial traffic was documented for 5 min at 37 °C. In (**H**), mitochondrial motility in a neuron treated with scramble siRNA. In (**I**), CRMP2 ablation with siRNA was accompanied by reduced mitochondrial traffic. In (**J**), a neuron with deleted CRMP2 and treated with 10 μM (S)-LCM for 7 days; mitochondrial traffic remained suppressed. In (**K**), the length of neuronal mitochondria in µm. In (**L**), fractions of moving organelles and mitochondria trafficking in retrograde and anterograde directions shown as percentage from total number of analyzed mitochondria. In (**K**,**L**), data are mean ± SD, * *p* < 0.05, ** *p* < 0.01, *** *p* < 0.001, N = 5 separate experiments with neurons from different platings. Green circles, neurons transfected with scramble siRNA; red circles, neurons transfected with anti-CRMP2 siRNA; blue circles, neurons transfected with anti-CRMP2 siRNA and treated with (S)-LCM. Data were analyzed by nonparametric Kruskal–Wallis ANOVA on ranks followed by Tukey test.

Here and in Figure 7E–G, we did not attempt to quantitatively assess mitochondrial morphology in neuronal somata, overcrowded with mitochondria; but, nevertheless, we presented images of mitochondria in the neuronal somata to illustrate changes in mitochondrial morphology. As we stated in the Materials and Methods, the dimensions of individual mitochondria were assessed with separate organelles positioned in neurites.

To test if alterations in mitochondrial motility and morphology in neurons are specifically linked to CRMP2, we tested the effect of CRMP2 genetic ablation on mitochondrial traffic and mitochondrial shape in cortical neurons in culture (Figure 7). We genetically ablated CRMP2 in cultured cortical neurons using a previously validated anti-CRMP2 siRNA delivered by electroporation [17]. Concurrently, neurons were transfected with cDNA encoding mito-eYFP to visualize mitochondria. CRMP2 was downregulated in neurons transfected with anti-CRMP2 siRNA, which could be found by mito-eYFP signal. In these experiments, we found that CRMP2 ablation correlated with increased fission (Figure 7E,F,I) and suppressed mitochondrial motility (Figure 7G,H,J). (S)-LCM was ineffective in CRMP2-depleted neurons (Figure 7), demonstrating (S)-LCM specificity toward CRMP2. Thus, ablation of CRMP2 recapitulated changes in mitochondrial motility and morphology induced by hyperphosphorylation of CRMP2 in AD neurons (Figure 6), validating specificity of (S)-LCM for CRMP2.

**Figure 8:**
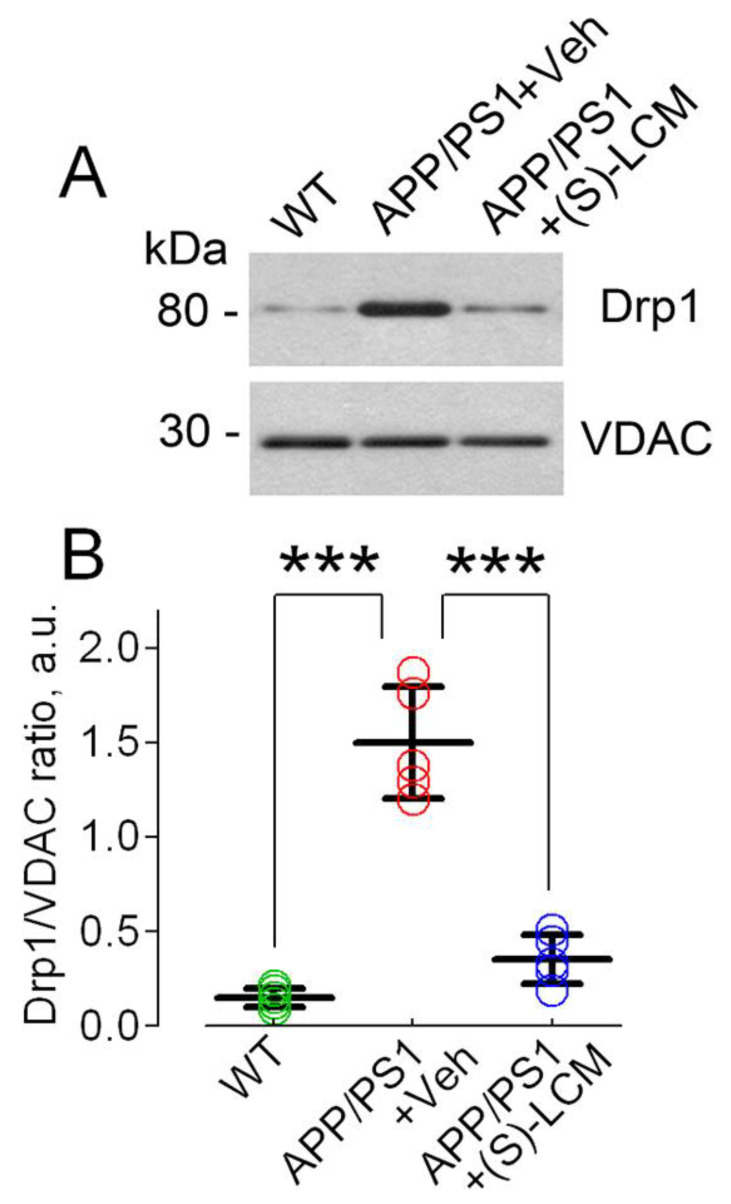
**Drp1 recruitment to mitochondria isolated from cultured cortical neurons from WT and APP/PS1 mice.** In (**A**), the representative immunoblots with anti-Drp1 and anti-VDAC1 antibodies. VDAC1 was a loading control. Where indicated, neurons from APP/PS1 mice were treated with a vehicle (Veh, 0.01% DMSO) or with 10 μM (S)-LCM for 7 days before the experiment. In (**B**), statistical summary of these experiments. The data are shown as a ratio of Drp1/VDAC1. Green circles, neurons from wild-type (WT) mice; red circles, neurons from AD mice treated with a vehicle; blue circles, neurons from AD mice treated with (S)-LCM.Data are mean ± SD, *** *p* < 0.001, N = 5 separate experiments with neurons from different platings. Data were analyzed by parametric one-way analysis of variance (ANOVA) with Holm–Sidak post-test. a.u., arbitrary units.

To better understand how changes occur in the shape of mitochondria in AD, we conducted experiments using mitochondria obtained from cultured cortical neurons. We observed the recruitment of Drp1 to mitochondria in cortical neurons from mice with the APP/PS1 gene mutation and found that Drp1 levels were higher in these cells compared to cells from wild-type mice (as shown in Figure 8). When we treated the cells with (S)-LCM at a concentration of 10 μM for 7 days before analysis, we observed a decrease in Drp1 recruitment to the mitochondria (as shown in Figure 8). This finding is consistent with our observation of decreased mitochondrial fragmentation (as shown in Figure 6).

### 3.4. CRMP2 and Cell Viability of AD Neurons

**Figure 9:**
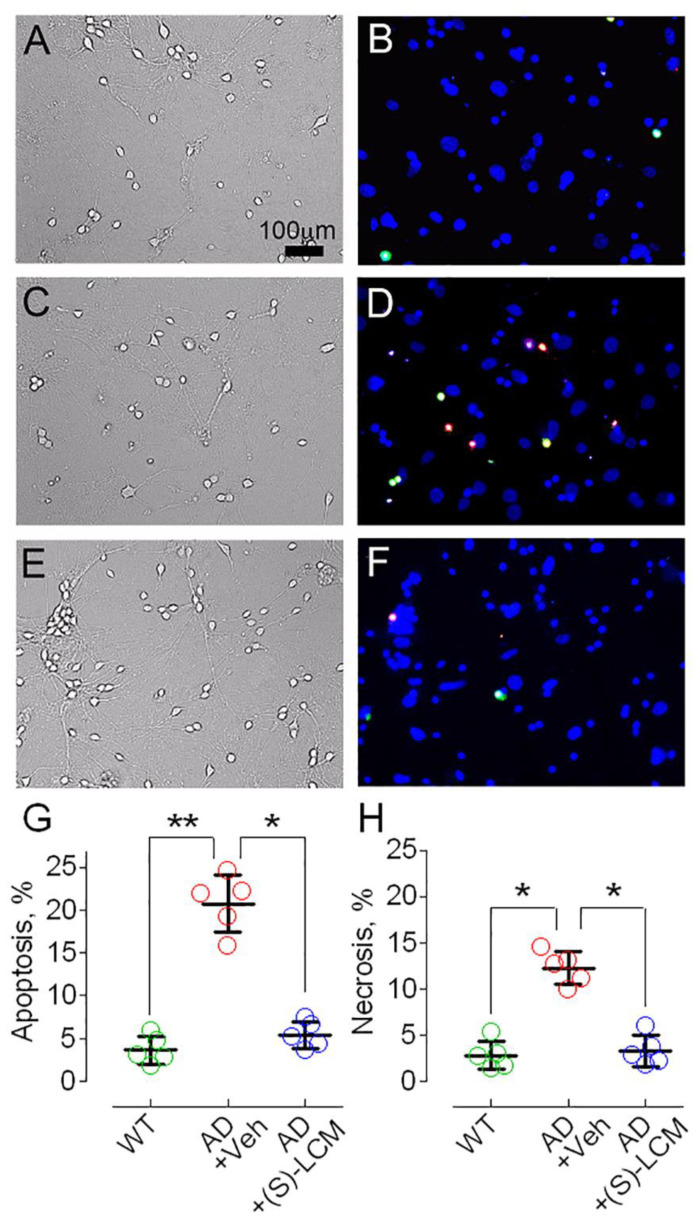
**Apoptotic and necrotic cell death in cortical neurons in culture from APP/PS1 (AD) mice and WT littermates (WT).** Neurons were cultured in vitro for 21 days and then stained with Hoechst 33342 (blue, nuclear marker), YO-PRO-1 (green, apoptosis marker), and propidium iodide (red, necrosis marker), respectively. (**A**,**B**), bright-field and fluorescent images of WT neurons. (**C**,**D**), bright-field and fluorescent images of AD neurons treated with a vehicle (Veh, 0.01% DMSO) for seven days before cell death assay. (**E**,**F**), phase-contrast bright-field and fluorescence images of AD neurons incubated with 10 µM (S)-LCM for seven days before cell death assay [18]. In (**G**,**H**), statistical summary of cell death in AD and WT neurons. Green circles, neurons from wild-type (WT) mice; red circles, neurons from AD mice treated with a vehicle; blue circles, neurons from AD mice treated with (S)-LCM. Data are mean ± SD, * *p* < 0.05, ** *p* < 0.01, N = 5 separate experiments with neurons from different platings. Data were analyzed by nonparametric Kruskal–Wallis ANOVA on ranks followed by Tukey test.

Abnormalities in mitochondrial morphology and motility could be disadvantageous for neurons. In our studies, cultured cortical neurons from APP/PS1 and WT mice had negligible cell death at 14 DIV. Therefore, we extended neuronal culturing until 21 DIV. At 21 DIV, neuronal cell death was evident in both AD and WT neurons. However, cultured cortical neurons from APP/PS1 mice were more predisposed to cell death comparing with cortical neurons from WT mice (Figure 9).

We also plotted our data as number of total cells analyzed, and number of apoptotic and necrotic cells (Supplementary Figure S8) This finding does not necessarily reflect neuronal cell death in vivo, but suggests that AD neurons in vitro are more stressed than neurons from WT animals. The exact mechanisms leading to cell death of cultured AD neurons are not yet clear. However, pre-treatment with (S)-LCM (10 μM in the growth medium for seven days before experiment) markedly protected AD neurons and improved their viability (Figure 9). Thus, (S)-LCM-induced preclusion of CRMP2 hyperphosphorylation at Ser 522 and Thr 509/514 correlated with preservation of mitochondrial motility and morphology and enhanced viability of AD mouse cortical neurons in culture.

## 4. Discussion

Alzheimer’s disease (AD) is a severe neurological condition that results in neuronal dysfunction and memory impairment [1,2]. The accumulation of β-amyloid plaques and neurofibrillary tangles in the brain is one of the most harmful pathological abnormalities observed in AD, leading to the degradation of neurons [3,4]. This neural degeneration can result in behavioral and cognitive deficits due to the malfunction and loss of synapses. Although much effort has been devoted to understanding the mechanisms underlying these neuronal dysfunctions and synaptic losses, our understanding is still incomplete. Unfortunately, there is no cure for AD, which is why it is crucial to continue research efforts aimed at discovering novel mechanisms contributing to AD pathology.

Continuous changes in mitochondrial motility and morphology (*mitochondrial dynamics*) play a critical role in maintaining mitochondrial health and supporting energy burdens in neurons [5]. Impairments in mitochondrial motility and morphology contribute to various neurodegenerative diseases such as Huntington’s and Alzheimer’s (AD) diseases [5,6,9,53,54]. It has been postulated that deficits in mitochondrial functions [11,12,13,14], oxidative stress, and synaptic dysfunction [55] are significant contributors to AD pathogenesis. Mitochondrial functions depend on a fission–fusion balance [56,57]. Increased fission leads to oxidative stress [58] and increased probability of apoptosis [59]. Mitochondrial fission is increased in AD and inhibition of fission is neuroprotective in AD mice [8,60,61,62,63,64]. Mitochondrial traffic in neurons is crucial for maintaining synaptic activity and eliminating damaged mitochondria [38]. In AD, mitochondrial traffic could be hindered by interaction of Aβ with proteins involved in mitochondrial motility [42,65] or by other as yet unknown mechanisms.

CRMP2 is an abundant phosphoprotein in the cytosol originally proposed to participate in axon guidance and neurite outgrowth through the Semaphorin 3A pathway [15,66,67]. CRMP2 performs its regulatory actions by interacting with different proteins [15,16] and these interactions regulate their activity and/or location [15]. CRMP2 interacts with proteins involved in regulating mitochondrial morphology such as Drp1 [17,18] and Mitofusin 2 (Figure 5) and with proteins involved in regulating mitochondrial motility such as Miro 2 [17,18], Kinesin 1 light chain (KLC1) [17,19], and Dynein [21]. Interaction of CRMP2 with its binding partners is modulated by CRMP2′s post-translational modifications such as phosphorylation [67]. Non-phosphorylated CRMP2 is functional and stimulates axon/neurite outgrowth while CRMP2 phosphorylation reduces its functional activity [24]. CRMP2 is phosphorylated at various sites by different kinases. Rho kinase phosphorylates CRMP2 at Thr 555 to trigger growth cone collapse [68,69]. The kinase Fyn from the src family phosphorylates Tyr 32 of CRMP2 and also results in neurite retraction and collapse of growth cones [70]. In addition, CRMP2 phosphorylation by GSK-3β kinase at Thr 509 and Thr 514 also cause neurite retraction and growth cone collapse [24,71]. Importantly, the kinase Cdk5 phosphorylates Ser 522 of CRMP2 and primes CRMP2 for further phosphorylation at Thr 509/514 by GSK-3β [25].

In our Western blotting experiments, we found CRMP2 bands with slightly different molecular weights. The bands with different molecular weights in the Western blots with postmortem human brain samples illustrated in Figure 1A and Figure 5 are most likely due to distinct isoforms of CRMP2, CRMP2A and CRMP2B, reported also by Mokhtar et al. [20]. They might also be a consequence of CRMP2 posttranslational alterations. Additionally, some of the bands might be due to partial CRMP2 degradation. Thus, all observed bands were utilized for densitometry. The reason for the variances in the bands of different sizes (Figure 1A) and why sometimes we can detect only one band with mouse cultured neurons (Figure 5) is not completely clear.

Recent studies have implicated CRMP2 as an emerging target in AD research [35]. CRMP2 is hyperphosphorylated in AD [20,32,33,34] and potentially represents a druggable target for therapeutic intervention [72]. Increased CRMP2 phosphorylation in AD mice occurs prior to pathology, suggesting that hyperphosphorylation of CRMP2 is an early incident in AD [32]. In line with this, cortical neurons from E14 mice cultured for 3 days and then exposed to a core toxic fragment of Aβ peptides (Aβ_25–35_) for another 3 days have a significant increase in CRMP2 phosphorylation [73], indicating that indeed CRMP2 hyperphosphorylation in cultured cortical neurons may occur very early following a short exposure to Aβ_25–35_.

While numerous studies have documented CRMP2 hyperphosphorylation in AD mice and human AD brains, the functional effects of CRMP2 hyperphosphorylation on the progression of AD remain unclear. CRMP2 hyperphosphorylation, increased mitochondrial fragmentation, and reduced mitochondrial traffic were reported in different AD mouse models, including APP/PS1 mice [8,10,32,42,74], suggesting a link between the CRMP2 phosphorylation state and abnormalities in mitochondrial motility and morphology in AD. These abnormalities in mitochondrial motility and morphology could potentially lead to defective mitochondrial bioenergetics in AD [11,12,13,14] and augmented neuronal cell death [8,75,76,77,78,79]. However, whether CRMP2 hyperphosphorylation is mechanistically linked to aberrant mitochondrial dynamics and reduced viability of AD neurons has not been investigated.

Our experiments with mouse cortical neurons in culture demonstrated that CRMP2, in a phosphorylation-dependent manner, interacts with Drp1, Miro 2, and Mitofusin 2. CRMP2 was hyperphosphorylated in cultured cortical neurons from double-transgenic APP/PS1 mice, a commonly used AD mouse model [42]. CRMP2 hyperphosphorylation was paralleled by a diminished interaction of CRMP2 with its binding partners, was involved in controlling mitochondrial motility and morphology, and was accompanied by diminished mitochondrial motility, augmented mitochondrial fragmentation, and increased neuronal cell death. Consequently, CRMP2 hyperphosphorylation could be associated with detrimental alterations in mitochondrial dynamics and reduced viability of neurons. A small molecule (S)-LCM, which selectively suppresses CRMP2 phosphorylation at Ser 522 and Thr 509/514 by Cdk5 and GSK-3β kinases [39,40], precluded CRMP2 hyperphosphorylation at these sites and rescued CRMP2’s protein–protein interactions. Interestingly, (S)-LCM dephosphorylated CRMP2, but not Drp1. This suggests that the effect of (S)-LCM most likely was due to CRMP2 dephosphorylation and not due to change in the Drp1 phosphorylation state. Intriguingly, genetic ablation of CRMP2 with anti-CRMP2 siRNA recapitulated the effect of CRMP2 hyperphosphorylation, leading to suppressed mitochondrial motility and excessive mitochondrial fragmentation. In this case, (S)-LCM was ineffective, confirming its specificity toward CRMP2. Thus, preventing CRMP2 phosphorylation with the small molecule (S)-LCM protected mitochondria from detrimental alterations in AD neurons. While we cannot completely exclude off-target effects of (S)-LCM, we are not aware of any of those off-target effects. Moreover, our experiments with CRMP2 downregulation and ineffective (S)-LCM treatment of neurons supports the lack of detectable (S)-LCM off-target effects.

The morphology of mitochondria is determined by a delicate balance between fission and fusion processes. However, our experiments were unable to distinguish between an increase in fission or a decrease in fusion. Nevertheless, previous research suggests that inhibiting Drp1 with Mdivi 1, a Drp1 inhibitor, can enhance mitochondrial morphology in AD neurons [62,80,81]. This observation strongly suggests that the increased fission mediated by Drp1 was the cause of augmented mitochondrial fragmentation in AD. Nevertheless, we plan to continue our investigation of the mechanisms by which CRMP2 could modulate mitochondrial morphology in AD and other neurodegenerative disorders.

Abnormal interaction of Drp1 with Aβ and phosphorylated Tau (pTau) in AD mice and in postmortem brain tissues from AD patients was linked to hyperactivation of Drp1 and augmented mitochondrial fission [82,83]. Based on our data, indicating CRMP2 binding to Drp1, it is possible that non-phosphorylated CRMP2 serves as *a molecular brake of fission*, hindering interaction of Drp1 with Aβ and pTau. However, this hypothesis requires additional investigation.

Drp1 is a cytosolic protein which is recruited to mitochondria to induce mitochondrial fission [37]. In our experiments, increased mitochondrial fragmentation in AD neurons was paralleled by diminished binding of CRMP2 to Drp1 and augmented Drp1 recruitment to mitochondria. This finding may explain increased fission of AD mitochondria. The beneficial effect of (S)-LCM on the shape of mitochondria correlated with the increased binding of CRMP2 to Drp1 and decreased Drp1 recruitment to mitochondria in cells treated with (S)-LCM. It is conceivable that CRMP2 interacting with Drp1 impedes Drp1 recruitment to mitochondria to avoid excessive mitochondrial fragmentation, whereas following hyperphosphorylation CRMP2 dissociates from Drp1 and, thereby, facilitates Drp1 recruitment to mitochondria.

In our study, we observed the interaction of CRMP2 with Miro 2 and Mitofusin 2. There is also a report of interaction between Mitofusin 2 and Miro 2 [84]. Mitofusin 2 is a key protein involved in mitochondrial fusion [5]. However, Mitofusin 2 is also involved in regulating mitochondrial motility. Mitofusin 2 is linked to a Miro/Milton complex and is necessary for mitochondrial traffic [84]. Miro 2 is an anchor protein, which together with Milton, links mitochondria to the molecular motors Kinesin 1 and Dynein [85,86,87]. It is conceivable that CRMP2 is a linker between Mitofusin 2 and the Miro/Milton complex. Alternatively, CRMP2 could be a linker between the Miro/Milton complex and kinesin motor because CRMP2 binds to Kinesin 1 [19] and Miro 2. CRMP2 hyperphosphorylation and/or deletion would disconnect the mitochondria from molecular motors and, consequently, reduce mitochondrial motility.

Our recent studies strongly suggest a phosphorylation-dependent participation of CRMP2 in regulating mitochondrial motility and morphology [17,18]. Our present study showed the effect of CRMP2 hyperphosphorylation on mitochondrial dynamics in AD neurons, but whether prevention of CRMP2 hyperphosphorylation can be neuroprotective in vivo and whether it can improve behavioral deficits in AD mice is not known yet. To date, only one study has examined the role of CRMP2 phosphorylation in a model of amyloidopathy [88]. By examining the effects of Aβ_25–35_ peptide, a core toxic fragment of Aβ proteins on behavioral and electrophysiological features in CRMP2 phosphorylation-deficient knock-in (*crmp2^ki/ki^*) mice, in which Ser 522 was replaced with Ala [89], this study revealed that Aβ_25–35_ induced impairments of cognitive memory and synaptic plasticity in WT mice but not in *crmp2^ki/ki^* mice [88]. It was also reported that CRMP2 is required in an early stage of memory consolidation [90], thereby providing a crucial link between CRMP2 and AD. These studies suggest that knock-in mice, which are resistant to the phosphorylation of CRMP2 by the kinases Cdk5 and GSK-3β that are activated in AD, show resilience to cognitive impairment caused by Aβ25–35 oligomers. Despite this, the precise functional implications of CRMP2 hyperphosphorylation in AD are not yet fully understood. Our current study sheds light on some potential mechanisms by which CRMP2 hyperphosphorylation may affect both neuronal mitochondria and the entire neuron. However, further research is necessary to investigate additional potential mechanisms of CRMP2-mediated functional changes in mitochondria associated with CRMP2 hyperphosphorylation in AD.

## Data Availability

Data are contained within the article or supplementary materials.

## Supplementary Materials

The following supporting information can be downloaded at: https://www.mdpi.com/article/10.3390/cells12091287/s1, Figure S1. The unedited images of immunoblots for Figure 1A. Figure S2. The unedited images of immunoblots for Figure 2A. Figure S3. The unedited images of immunoblots for Figure 3A. Figure S4. The unedited images of immunoblots for Figure 4D. H, homogenate; C, cytosol; Mtc, mitochondria. Figure S5. The unedited images of immunoblots for Figure 5. Figure S6. **(S)-LCM does not alter mitochondrial motility and morphology in cultured cortical neurons from WT mice.** In (**A**–**D**), representative images of mitochondria in cultured cortical neurons (12-14 DIV) visualized with mito-eYFP and imaged with a spinning-disk confocal microscopy. Mitochondrial morphology was analyzed using 3D reconstruction of serial images (z-stacks). In *Insets*, mitochondria are demonstrated at doubled magnification. In (**A**), neuron from **WT** mice, these neurons were treated with a vehicle (Veh, 0.01% DMSO) for 7 days before imaging; in (**B**), **WT** neuron incubated with 10 μM (S)-LCM for 7 days before imaging. In (**C**,**D**), mitochondrial traffic in a neurite of **WT** neuron treated with a vehicle (Veh, 0.01% DMSO) for 7 days before imaging (**C**), and **WT** neuron treated with 10 μM (S)-LCM for 7 days before imaging (**D**). Vertical traces represent immobile mitochondria, angled traces represent moving organelles. The bands with mitochondrial fluorescence images demonstrate the organelles at the beginning of experiment. Mitochondrial motility was recorded for 5 min at 37 °C. In (**E**), the length of neuronal mitochondria in µm. The shape (length) of individual organelles was assessed with single organelles positioned in neurites. One hundred randomly selected mitochondria from 10-12 neurons from three different platings were assessed employing Imaris Measurement Pro 6.4 software (Bitplane, South Windsor, CT, USA) [17,18,49]. In (**F**), fractions of total moving mitochondria and mitochondria moving in retrograde and anterograde directions shown as percentage from total number of mitochondria. Data are mean ± SD, N = 5 separate experiments with neurons from different platings. Data were analyzed by nonparametric Kruskal–Wallis ANOVA on ranks followed by Tukey test. Figure S7. The unedited images of immunoblots for Figure 8A. Figure S8. **Apoptotic and necrotic cell death in cultured cortical neurons from APP/PS1 (AD) mice and WT littermates (WT): cell count analysis.** Neurons were plated on the glassbottom 35 mm Petri dishes and cultured in vitro for 21 days and then stained with Hoechst 33342 (nuclear marker), YO-PRO-1 (apoptosis marker), and propidium iodide (necrosis marker), respectively. Cultured neurons from 5 different platings of three different types of neuronal cultures were used: (i) **WT** neurons, (ii) **AD** neurons treated with a vehicle (Veh, 0.01% DMSO) for 7 days prior to cell death analysis, and (iii) **AD** neurons treated with 10 µM (S)-LCM for 7 days prior to cell death analysis. With each cell culture, neurons were counted in a blind manner in two fields of view (each with 45–70 cells) and it was repeated with three different Petri dishes per each type of neuronal cultures. Total numbers of cells analyzed in each type of neuronal cultures were 1737 (**WT**), 1763 (**AD** + Veh), 1742 (**AD** + (S)-LCM). The data were plotted as the total number of analyzed cells (red symbols), Apoptotic (**A**) and Necrotic (**B**) cells per each type of neuronal cultures. Data are mean ± SD. *** *p* < 0.001, N = 5 separate experiments with neurons from different platings. Table S1. Main characteristics of human postmortem brain samples.
