# Supplementary material for: CRMP2 Participates in Regulating Mitochondrial Morphology and Motility in Alzheimer’s Disease
Source: Cells. 2023 Apr 29;12(9):1287. doi: 10.3390/cells12091287 (PMC10177167; doi:10.3390/cells12091287)
Supplement: Supplementary file 1 [file cells-12-01287-s001.zip › cells-2313543-SI.pdf]

SUPPLEMENTAL MATERIALS

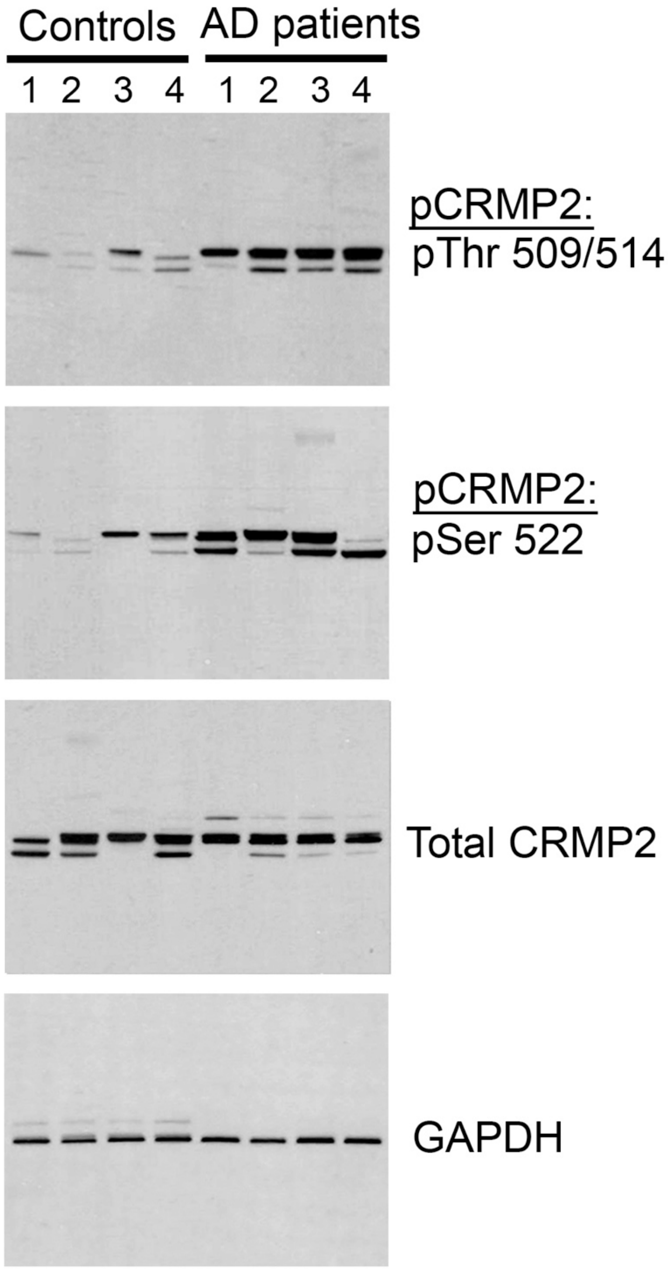

Figure S1. The unedited images of immunoblots for Figure 1A.

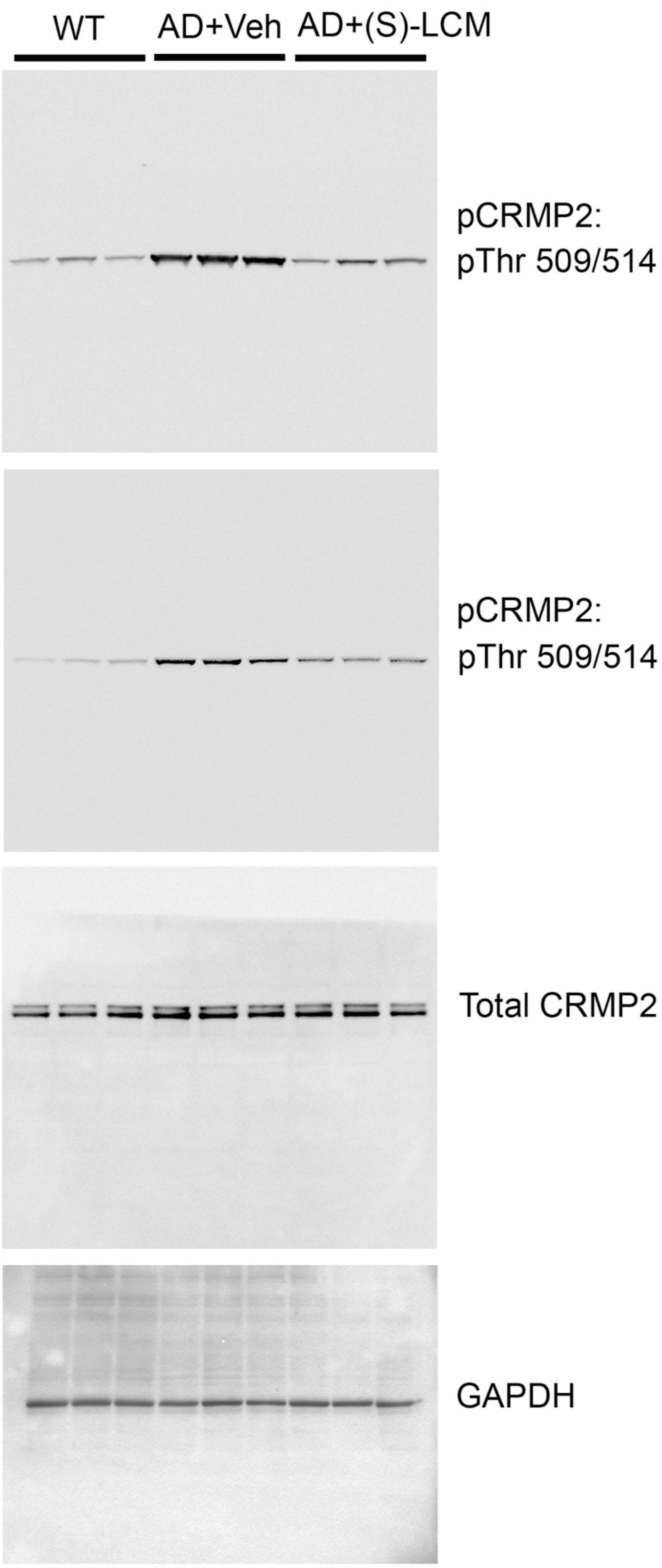

Figure S2. The unedited images of immunoblots for Figure 2A.

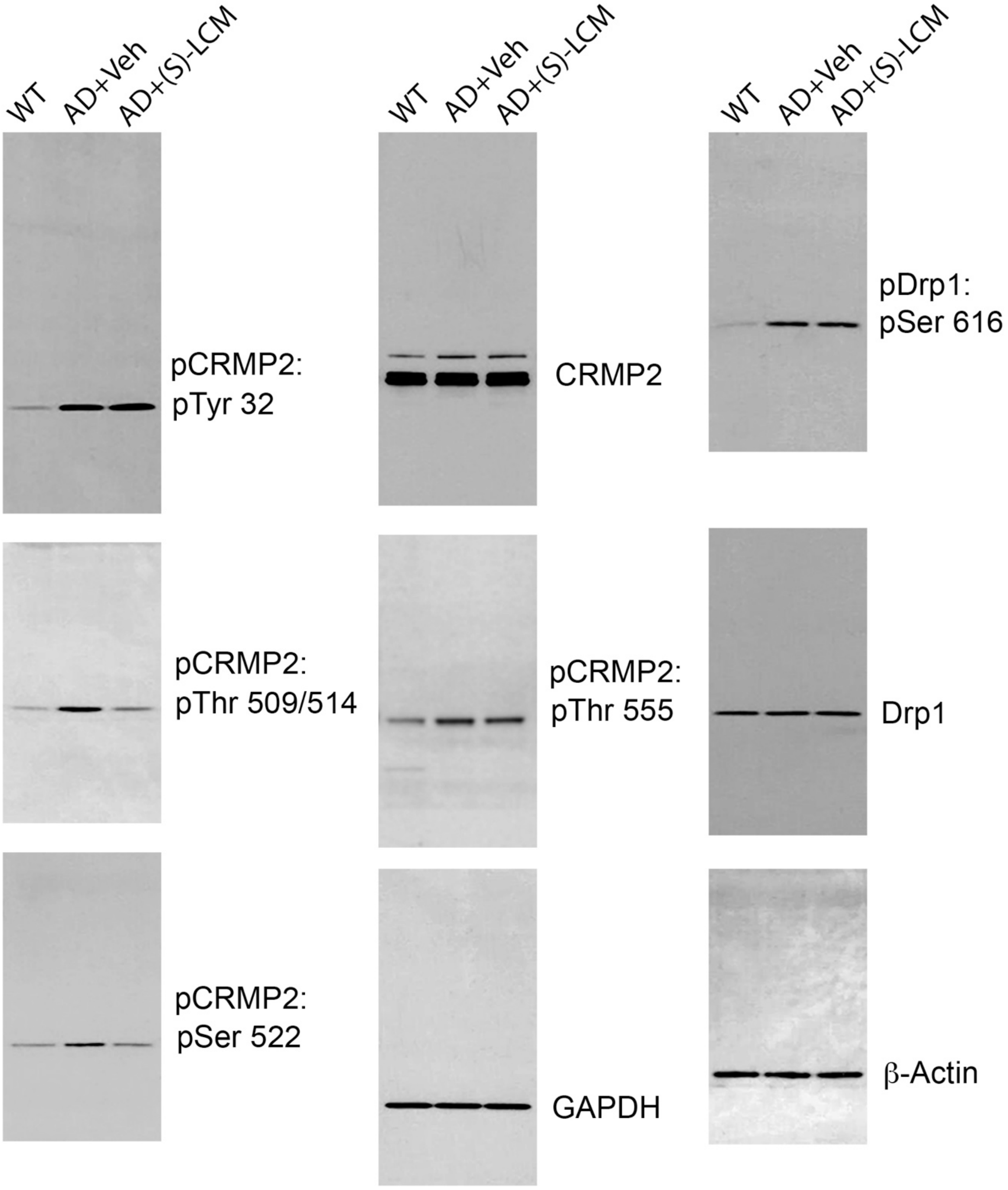

Figure S3. The unedited images of immunoblots for Figure 3A.

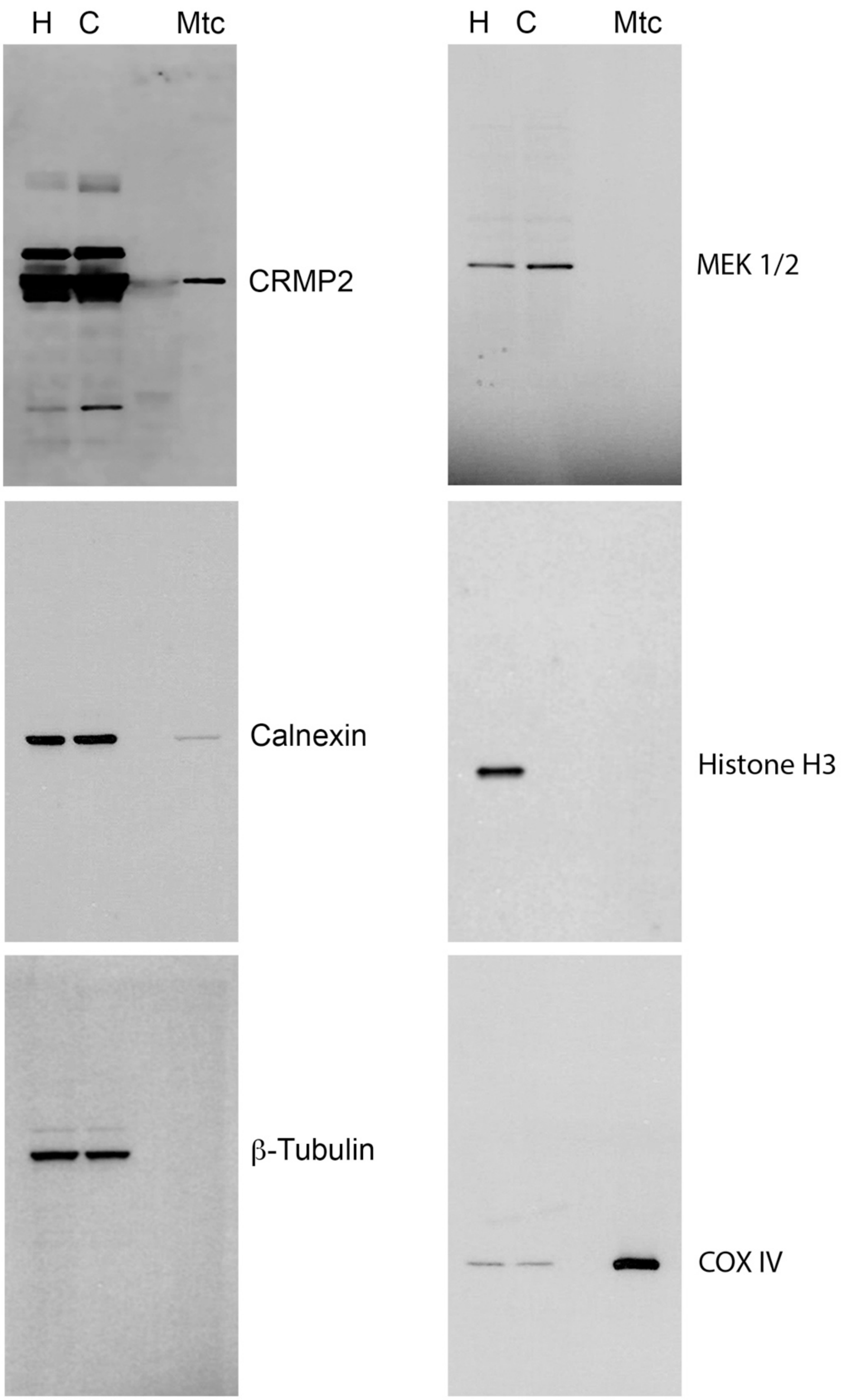

Figure S4. The unedited images of immunoblots for Figure 4D. H, homogenate; C, cytosol; Mtc, mitochondria.

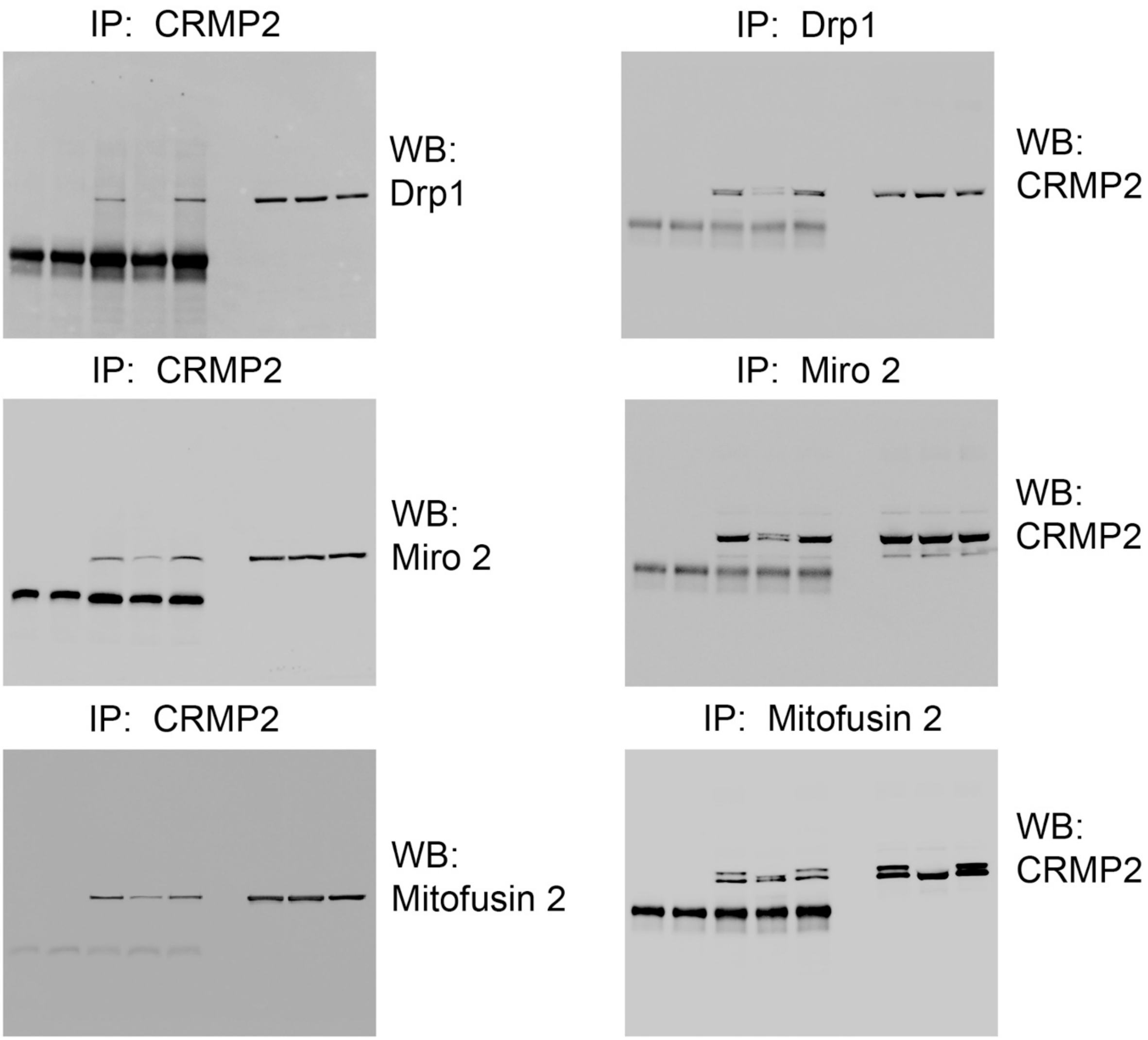

Figure S5. The unedited images of immunoblots for Figure 5.

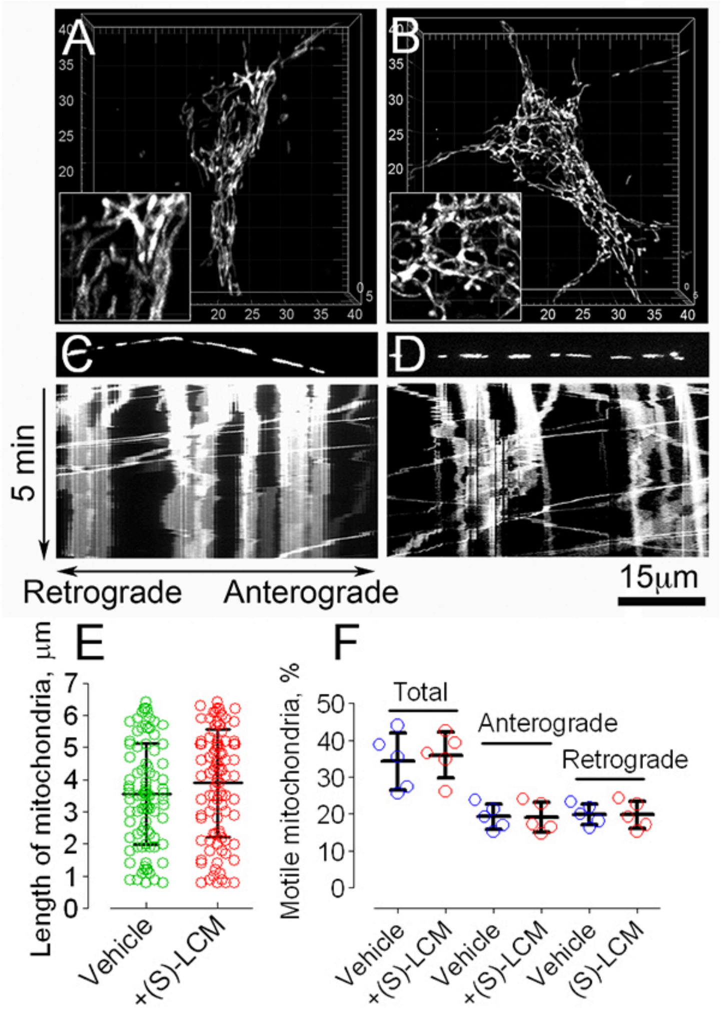

Figure S6. **(S)-LCM does not alter mitochondrial morphology and motility in cultured cortical neurons from WT mice.** In **A**-**D**, representative images of mitochondria in cultured cortical neurons (12-14 DIV) visualized with mito-eYFP and imaged with a spinning-disk confocal microscopy. Mitochondrial morphology was analyzed using 3D reconstruction of serial images (z-stacks). In *Insets*, mitochondria are demonstrated at extra 2x magnification. In **A**, neuron from **WT** mice, these neurons were treated with a vehicle (Veh, 0.01% DMSO) for 7 days before imaging; in **B**, **WT** neuron incubated with 10μM (S)-LCM for 7 days before imaging. In **C** and **D**, mitochondrial traffic in a neurite of **WT** neuron treated with a vehicle (Veh, 0.01% DMSO) for 7 days before imaging (**C**), and **WT** neuron treated with 10μM (S)-LCM for 7 days before imaging (**D**). Vertical traces show immobile mitochondria, angled traces indicate trafficking organelles. The strips with fluorescent images of mitochondria demonstrate the organelles at the beginning of recordings. Mitochondrial motility was recorded for 5 minutes at 37ºC. In **E**, the length of neuronal mitochondria in µm. The length of individual mitochondria was assessed with individual organelles positioned in neuronal processes. One hundred randomly selected mitochondria from 10-12 neurons from 3 different platings were assessed employing Imaris Measurement Pro 6.4 software (Bitplane, South Windsor, CT) [17,18,49] . In **F**, fractions of trafficking mitochondria and mitochondria moving in anterograde and retrograde directions expressed as percentage from total number of mitochondria. Data are mean±SD, N=5 separate experiments with neurons from different platings. Data were analyzed by nonparametric Kruskal-Wallis ANOVA on Ranks followed by Tukey test.

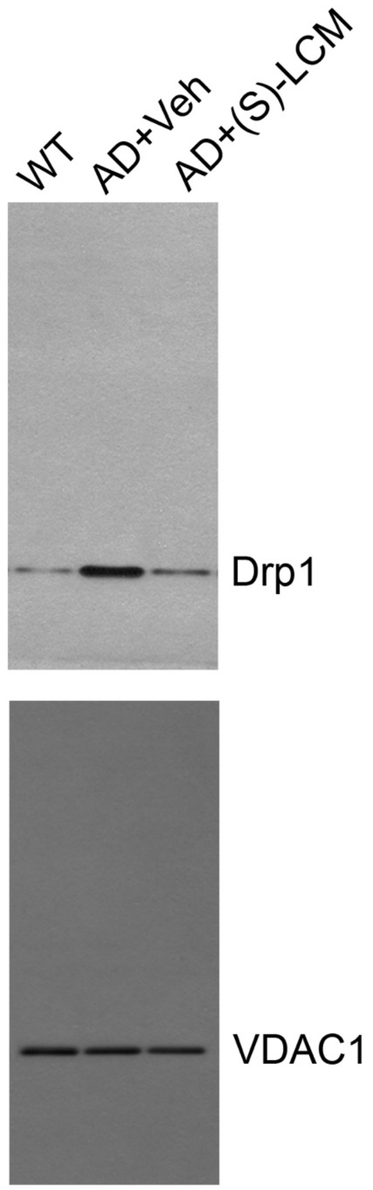

Figure S7. The unedited images of immunoblots for Figure 8A.

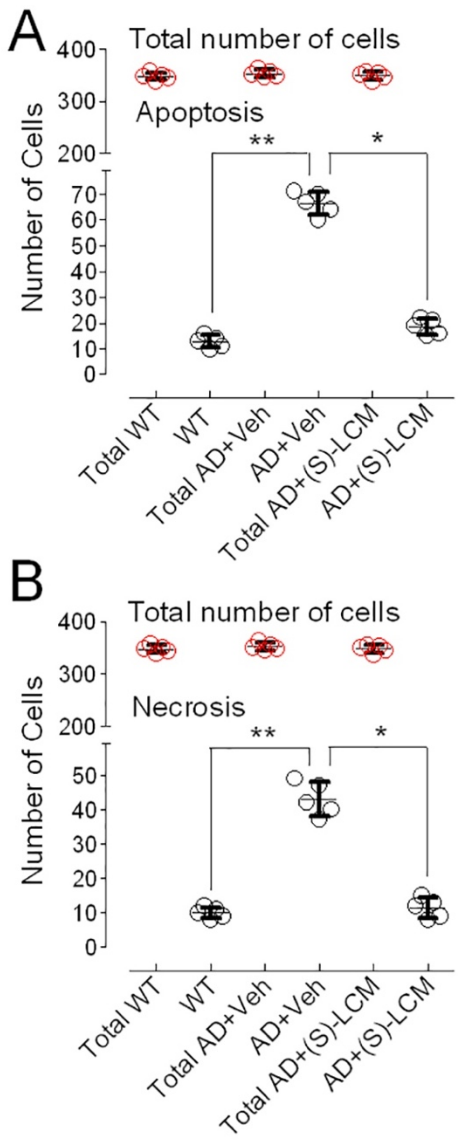

Figure S8. **Apoptotic and necrotic cell death in cultured cortical neurons from APP/PS1 (AD) mice and WT littermates (WT): cell count analysis.** Neurons were plated on the glass bottom 35 mm Petri dishes and cultured *in vitro* for 21 day and then stained with Hoechst 33342 (nuclear marker), YO-PRO-1 (apoptosis marker), and propidium iodide (necrosis marker), respectively. Cultured neurons from 5 different platings of three different types of neuronal cultures were used: (i) **WT** neurons, (ii) **AD** neurons treated with a vehicle (Veh, 0.01% DMSO) for 7 days prior to cell death analysis, and (iii) **AD** neurons treated with 10μM (S)-LCM for 7 days prior to cell death analysis. With each cell culture, neurons were counted in a blind manner in two fields of view (each with 45-70 cells) and it was repeated with three different Petri dishes per each type of neuronal cultures. Total numbers of cells analyzed in each type of neuronal cultures were 1737 (**WT**), 1763 (**AD**+Veh), 1742 (**AD**+(S)-LCM). The data were plotted as the Total number of analyzed cells (red symbols), Apoptotic (**A**) and Necrotic (**B**) cells per each type of neuronal cultures. Data are mean±SD. **p*<0.05, ***p*<0.01, Kruskal-Wallis ANOVA on Ranks (SigmaPlot 15.1), N=5 separate experiments with neurons from different platings.

Table S1.

Main characteristics of human postmortem brain samples

Sample ID#	Assigned #	Age	Postmortem Interval (hours)	Race	Sex	Brain Region
**Control Unaffected Individuals**						
583253	1	88	25	W	F	Pre-frontal cortex
13571	2	89	4	W	F	Pre-frontal cortex
893540	3	85	9	W	M	Pre-frontal cortex
660286	4	71	12	W	M	Pre-frontal cortex
**AD Patients**						
1018	1	85	12	W	F	Pre-frontal cortex
1312	2	87	7	W	F	Pre-frontal cortex
1252	3	78	9	W	M	Pre-frontal cortex
1212	4	72	18	W	F	Pre-frontal cortex
